# Supplementary material for: A scalable and cGMP-compatible autologous organotypic cell therapy for Dystrophic Epidermolysis Bullosa
Source: Nat Commun. 2024 Jul 11;15:5834. doi: 10.1038/s41467-024-49400-z (PMC11239819; doi:10.1038/s41467-024-49400-z)
Supplement: Supplementary file 6 — Supplementary Data 3 [file 41467_2024_49400_MOESM6_ESM.zip › Supplementary Data 3/57_offtarget_sites/CO1-173_2KB/patient3_2.chr19_1730409_1730431.2KB.pdf]

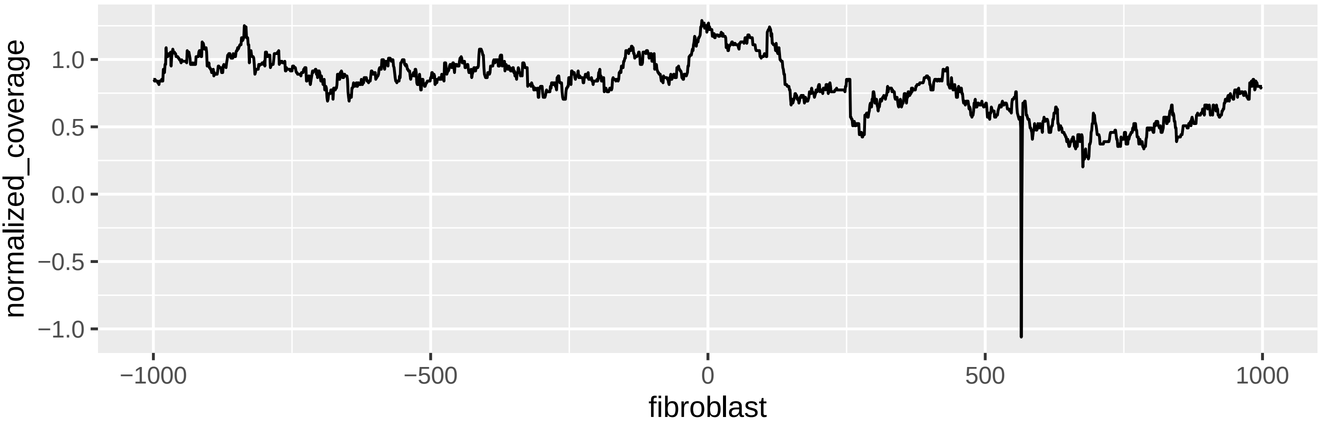

normalized_coverage
1.0
0.5
0.0
−0.5
−1.0
−1000
−500
0
500
1000
fibroblast

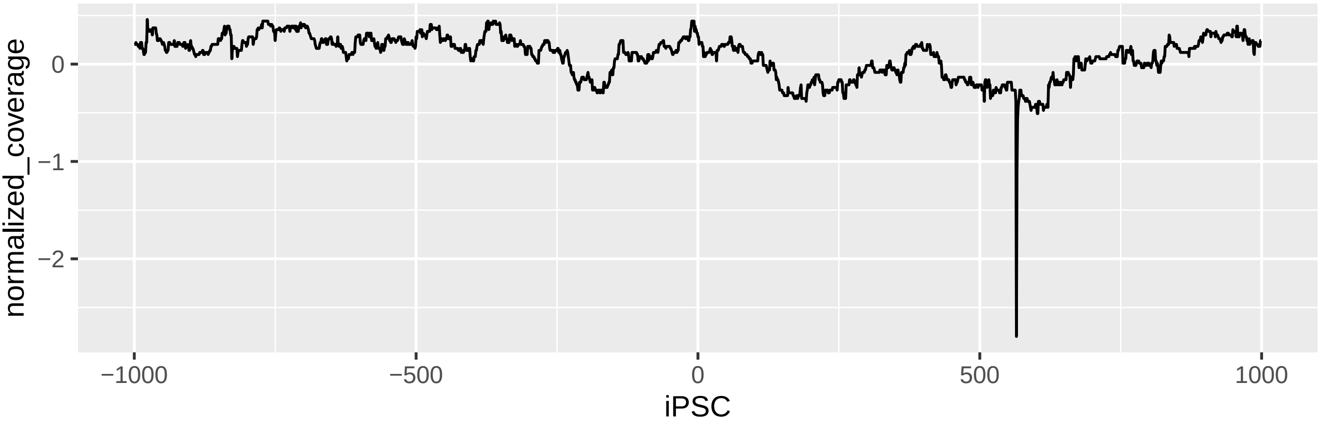

normalized_coverage
0
−1
−2
−1000
−500
0
500
1000
iPSC

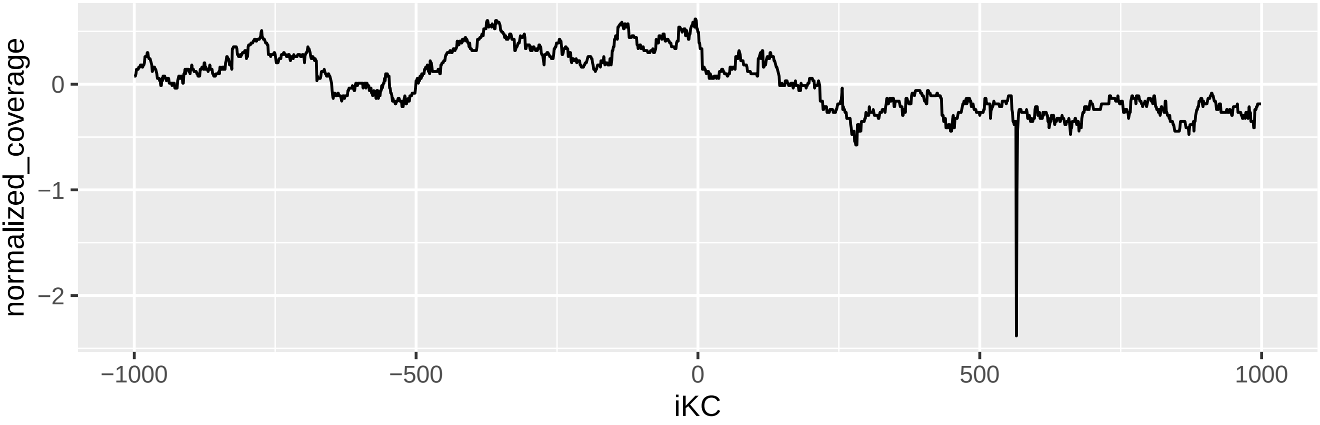

normalized_coverage
0
−1
−2
−1000
−500
0
500
1000
iKC
